# Renin-Angiotensin System Inhibition in Patients With Myocardial Injury Complicating Transcatheter Aortic Valve Replacement

**DOI:** 10.1016/j.jacadv.2024.101212

**Published:** 2024-08-16

**Authors:** Daijiro Tomii, Dik Heg, Jonas Lanz, Masaaki Nakase, Daryoush Samim, Stefan Stortecky, David Reineke, Stephan Windecker, Thomas Pilgrim

**Affiliations:** a Department of Cardiology, Inselspital, Bern University Hospital, University of Bern, Bern, Switzerland; b Department of Clinical Research, University of Bern, Switzerland; c Department of Cardiac Surgery, Inselspital, Bern University Hospital, University of Bern, Bern, Switzerland

**Keywords:** aortic stenosis, periprocedural myocardial injury, renin-angiotensin system inhibitors, transcatheter aortic valve replacement, Valve Academic Research Consortium

## Abstract

**Background:**

Periprocedural myocardial injury impacts clinical outcome after transcatheter aortic valve replacement (TAVR). The optimal medical management strategy for TAVR-related periprocedural myocardial injury has not been established.

**Objectives:**

The authors aimed to investigate the prognostic association of renin-angiotensin system (RAS) inhibitors in patients with periprocedural myocardial injury after TAVR.

**Methods:**

In a prospective TAVR registry, patients were retrospectively stratified according to Valve Academic Research Consortium (VARC)-3 periprocedural myocardial injury and RAS inhibitor prescription after TAVR. The main outcomes of interest were prevalence of myocardial injury and cardiovascular death. Logistic and Cox proportional hazards regression were used to analyze outcomes of interest.

**Results:**

Among 2,083 eligible patients undergoing TAVR between August 2007 and June 2023, 283 patients (13.8%) developed VARC-3 periprocedural myocardial injury. RAS inhibitors were prescribed in 197 patients (70%) with periprocedural myocardial injury and in 1,251 patients (71.2%) without injury. Compared with patients without periprocedural myocardial injury, patients with myocardial injury had an increased risk of cardiovascular death at 1 year (HR_adjusted:_ 2.08; 95% CI: 1.39-3.11). The use of RAS inhibitors after TAVR was associated with a reduced risk of cardiovascular death in patients with and without periprocedural myocardial injury (HR_adjusted:_ 0.46; 95% CI: 0.22-0.95, and HR_adjusted:_ 0.44; 95% CI: 0.30-0.65, respectively).

**Conclusions:**

One out of 7 patients undergoing TAVR experienced periprocedural myocardial injury. VARC-3 periprocedural myocardial injury was associated with a 2-fold increased risk of cardiovascular death at 1 year after TAVR. The favorable association of RAS inhibitor prescription was consistent in patients with and without periprocedural myocardial injury. (SwissTAVI Registry; NCT01368250)

Transcatheter aortic valve replacement (TAVR) continues to expand as less invasive treatment of aortic stenosis (AS) allowing for a faster recovery compared to surgical aortic valve replacement.^1^^,^^2^ Elevations in cardiac biomarkers are common in patients undergoing TAVR; however, the prognostic implications of periprocedural myocardial injury remain unclear. Definitions of myocardial injury following TAVR have been subject to change.^3^, ^4^, ^5^, ^6^, ^7^ In the recently updated Valve Academic Research Consortium (VARC-3) document, periprocedural myocardial injury has been defined specifically using a higher biomarker threshold compared to previous definitions. A recent study suggests that the definition according to the VARC-3 consensus document more accurately reflects the incidence and prognostic impact of periprocedural myocardial injury.^8^^,^^9^ However, the optimal medical management strategy for patients who develop periprocedural myocardial injury after TAVR is still unclear.

Renin-angiotensin system (RAS) inhibition is an established medical therapy that attenuates myocardial hypertrophy and fibrosis, and improves clinical outcomes in patients with heart failure, and those with acute or chronic coronary syndromes.^10^ Although observational and registry data support the use of RAS inhibitors after AVR in patients with severe AS,^11^, ^12^, ^13^, ^14^, ^15^ there are limited data on the association between RAS inhibitor prescription and clinical outcomes in patients who develop periprocedural myocardial injury after TAVR. Therefore, in the present study, we sought to investigate the prognostic association of RAS inhibitors on VARC-3 periprocedural myocardial injury in patients with severe AS undergoing TAVR.

## Methods

### Study design and population

All patients undergoing TAVR for severe symptomatic AS at Bern University Hospital (Bern, Switzerland) are enrolled into an institutional prospective registry, which forms part of the nationwide SwissTAVI registry (NCT01368250).^16^ The present analysis included consecutive patients who underwent transfemoral TAVR for native severe AS between August 2007 and June 2023. For the purpose of this study, patients with missing data required for the assessment of VARC-3 periprocedural myocardial injury, missing information on RAS inhibitor prescription at discharge after TAVR, or patients with in-hospital death were excluded. The registry was approved by the Bern cantonal ethics committee, and patients provided written informed consent for participation.

### Data collection and clinical endpoints

All baseline clinical, procedural, and follow-up data were prospectively recorded in a dedicated database, held at the Clinical Trials Unit of the University of Bern. Echocardiographic measurements were re-evaluated by dedicated imaging specialists and integrated into the database. Clinical follow-up data at 30 days and 1 year after TAVR were obtained by standardized interviews, documentation from referring physicians, and hospital discharge summaries as previously described.^17^ All adverse events were systematically collected and adjudicated by a dedicated clinical event committee on the basis of the VARC criteria applicable at the time of the procedure.^3^^,^^8^^,^^18^ Technical success was defined according to the VARC-3 definition and adjudicated retrospectively as described previously.^8^^,^^19^

### VARC-3 periprocedural myocardial injury and prescription of ras inhibitors

Periprocedural myocardial injury according to the VARC-3 definition was adjudicated retrospectively on the basis of the following detailed, systematically collected information: 1) the peak creatine kinase-myocardial band (CK-MB) measured within 48 hours of TAVR ≥10 times the upper limit of normal in patients with normal baseline CK-MB; 2) the peak cardiac troponin measured within 48 hours of TAVR ≥70 times the upper limit of normal in patients with normal baseline cardiac troponin; or 3) an absolute increment equal to those levels from the most recent preprocedure level within 48 hours of TAVR plus new persistent left bundle branch block in patients with elevated baseline CK-MB or cardiac troponin.^8^ Cardiac troponin and CK-MB levels were measured within 12 hours post-TAVR, and in case of elevated levels, repeat measurements were performed every 6 to 8 hours to assess peak post-TAVR levels. CK-MB and cardiac troponin measurements were performed up to August 2010 using the fourth-generation Elecsys assays and thereafter using the fifth-generation Elecsys assays (both Roche Diagnostics). Based on the 99th percentile and a coefficient of variation of ≤10%, the upper reference limits for CK-MB, cardiac troponin T, and high-sensitivity troponin T levels were 3.6 μg/L, 10 ng/L, and 14 ng/L, respectively.^4^^,^^20^ Twelve-lead electrocardiograms were recorded at baseline, immediately after the procedure, and at hospital discharge. Repeat measurements were obtained in case of new-onset left bundle branch block.^21^

Patients were stratified according to the prescription of RAS inhibitors at discharge, including angiotensin-converting enzyme inhibitors, angiotensin II receptor blockers, or angiotensin receptor-neprilysin inhibitors at the time of discharge after TAVR.

### Outcomes of interest

The main objectives of the present study were: 1) to document the incidence of VARC-3 periprocedural myocardial injury after TAVR; 2) to report the rate of RAS inhibitor prescription according to the development of periprocedural myocardial injury; and 3) to evaluate clinical outcomes according to the presence of periprocedural myocardial injury and prescription of RAS inhibitors.

### Statistical analysis

Categorical data are represented as frequencies and percentages, and the differences between groups were evaluated with the chi-square test or Fisher’s exact test. Continuous variables are presented as mean ± SD and were compared between groups using Student’s *t*-test. Univariable and multivariable logistic regression analysis was performed to investigate the association of RAS inhibitors on the development of VARC-3 periprocedural myocardial injury. Rate ratios with 95% CIs from Poisson regressions were provided where appropriate. Cumulative time-to-event curves were constructed using the Kaplan-Meier method. Cox proportional hazards models were used to calculate crude or adjusted HRs and 95% CIs. Multivariable adjustment was performed with predefined baseline variables potentially related to clinical outcomes including age, sex, body mass index, and Society of Thoracic Surgeons Predicted Risk of Mortality (STS-PROM). We assessed cardiovascular mortality using a full factorial design that allows the main effects of factors and their interactions to be examined simultaneously while controlling for type I error.^22^^,^^23^ In addition, we performed a sensitivity analysis including only patients with technical success since it was anticipated that procedural adverse events may influence the development of periprocedural myocardial injury and the prescription of RAS inhibitors, leading to a bias in clinical outcomes. All statistical tests were 2-sided and *P* values of <0.05 were considered significant. Statistical analyses were performed using Stata, version 15.1 (StataCorp).

## Results

### Study population and baseline characteristics

**Figure 1:**
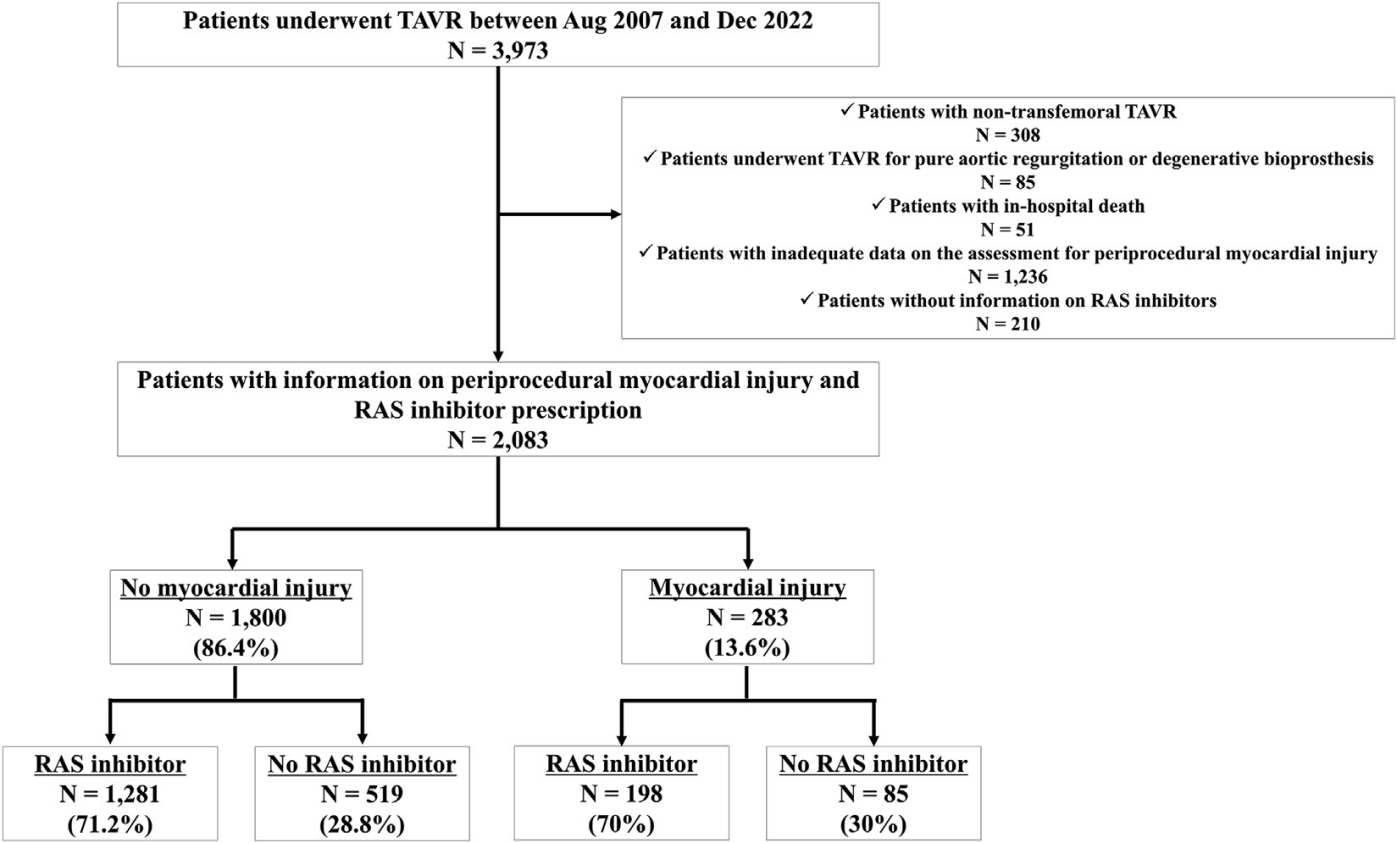
**Study Flowchart** RAS = renin-angiotensin system; TAVR = transcatheter aortic valve replacement.

Among 3,973 consecutive patients enrolled into the prospective Bern TAVI registry, 2,083 patients met the inclusion criteria (Figure 1). Of these, 283 patients (13.6%) experienced VARC-3 periprocedural myocardial injury after TAVR. RAS inhibitors were prescribed in 198 patients (70%) with periprocedural myocardial injury and in 1,281 patients (71.2%) without injury at discharge after TAVR (Central Illustration).

**Central Illustration:**
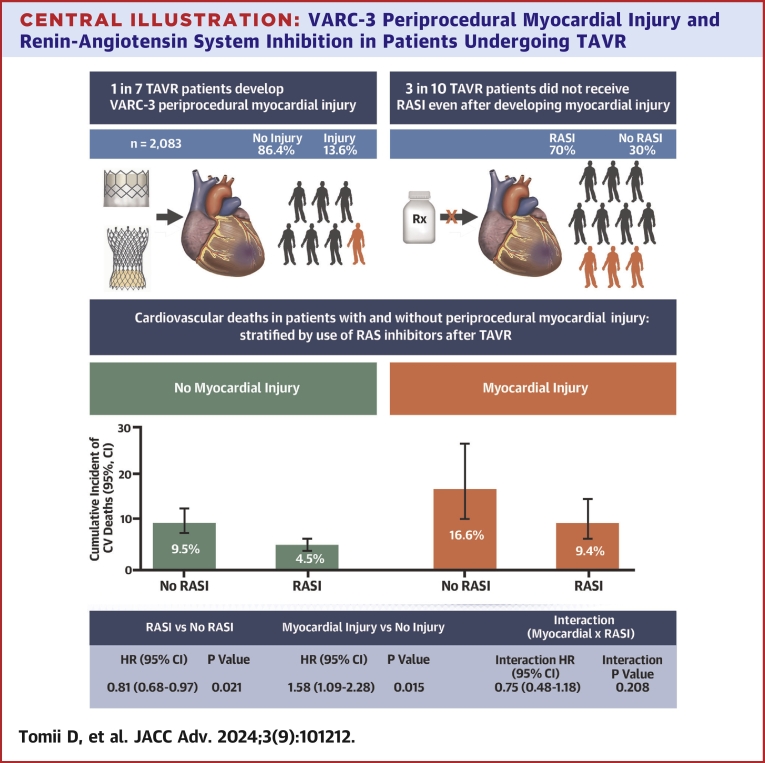
**VARC-3 Periprocedural Myocardial Injury and Renin-Angiotensin System Inhibition in Patients Undergoing TAVR** Incidence of VARC-3 periprocedural myocardial injury in patients undergoing TAVR and prescription rate of RAS inhibitors in patients who developed periprocedural myocardial injury (Upper). Full factorial model for cardiovascular death by VARC-3 myocardial injury and RAS inhibitor prescription After TAVR (Lower). Abbreviations as in Figures 1 and 2.

Baseline and procedural characteristics according to VARC-3 periprocedural myocardial injury are shown in Table 1. Overall, 1,045 patients (50.2%) were female, the mean age of the cohort was 82.3 ± 6.0 years, and the STS-PROM was 5.1% ± 3.7%. There was no relevant difference in baseline characteristics between patients with and without periprocedural myocardial injury. In particular, prevalence of coronary artery disease and previous myocardial infarction were comparable in patients with versus without periprocedural myocardial injury. TAVR was performed under general anesthesia in 11.2% of patients without significant difference between groups. Periprocedural myocardial injury was particularly common among patients with conversion to surgery (3.9% vs 1.0%; *P* = 0.001).

**Table 1:** Baseline and Procedural Characteristics According to VARC-3 Periprocedural Myocardial Injury and RAS Inhibitor Prescription at Discharge

	Overall Population				Patients Without Myocardial Injury			Patients With Myocardial Injury		
	All Patients (N = 2,083)	Patients Without Myocardial Injury (n = 1,800)	Patients With Myocardial Injury (n = 283)	*P* Value	RAS Inhibitors (n = 1,281)	No RAS Inhibitors (n = 519)	*P* Value	RAS Inhibitors (n = 198)	No RAS Inhibitors (n = 85)	*P* Value
Age, y	82.3 ± 6.0	82.3 ± 6.0	82.0 ± 6.1	0.402	82.3 ± 5.9	82.3 ± 6.3	0.995	82.0 ± 5.9	82.1 ± 6.7	0.883
Female	1,045 (50.2%)	893 (49.6%)	152 (53.7%)	0.202	630 (49.2%)	263 (50.7%)	0.568	96 (48.5%)	56 (65.9%)	0.009
Body mass index, kg/cm^2^	26.7 ± 5.3	26.7 ± 5.3	26.5 ± 5.6	0.549	26.9 ± 5.3	26.3 ± 5.2	0.020	26.5 ± 5.0	26.6 ± 6.8	0.968
STS-PROM, %	5.1 ± 3.7	5.1 ± 3.7	5.3 ± 4.0	0.342	5.1 ± 3.6	5.0 ± 3.7	0.630	5.4 ± 4.0	5.3 ± 4.0	0.925
NYHA functional class III or IV	1,416 (68.0%)	1,216 (67.6%)	200 (70.7%)	0.337	880 (68.8%)	336 (64.7%)	0.107	136 (68.7%)	64 (75.3%)	0.319
Urgent TAVR	57 (2.7%)	46 (2.6%)	11 (3.9%)	0.236	34 (2.7%)	12 (2.3%)	0.744	10 (5.1%)	1 (1.2%)	0.182
Systolic blood pressure <100 mm Hg	324 (19.1%)	279 (18.9%)	45 (20.4%)	0.646	209 (19.9%)	70 (16.5%)	0.162	29 (19.2%)	16 (22.9%)	0.591
Comorbidities										
Hypertension	1,808 (86.8%)	1,565 (86.9%)	243 (85.9%)	0.637	1,155 (90.2%)	410 (79.0%)	<0.001	180 (90.9%)	63 (74.1%)	0.001
Diabetes mellitus	562 (27.0%)	493 (27.4%)	69 (24.4%)	0.313	365 (28.5%)	128 (24.7%)	0.103	53 (26.8%)	16 (18.8%)	0.176
CKD (eGFR <60 mL/min/1.73 m^2^)	1,417 (68.2%)	1,226 (68.3%)	191 (68.0%)	0.945	866 (67.8%)	360 (69.5%)	0.502	132 (67.0%)	59 (70.2%)	0.676
Coronary artery disease	1,237 (59.4%)	1,065 (59.2%)	172 (60.8%)	0.649	788 (61.5%)	277 (53.4%)	0.002	128 (64.6%)	44 (51.8%)	0.047
Previous myocardial infarction	302 (14.5%)	255 (14.2%)	47 (16.6%)	0.276	200 (15.6%)	55 (10.6%)	0.006	38 (19.2%)	9 (10.6%)	0.083
Atrial fibrillation	734 (35.2%)	639 (35.5%)	95 (33.6%)	0.547	445 (34.7%)	194 (37.4%)	0.302	65 (32.8%)	30 (35.3%)	0.683
Peripheral artery disease	186 (8.9%)	163 (9.1%)	23 (8.1%)	0.736	117 (9.1%)	46 (8.9%)	0.928	19 (9.6%)	4 (4.7%)	0.236
Echocardiography										
Aortic valve area, cm^2^	0.74 ± 0.23	0.74 ± 0.23	0.71 ± 0.24	0.042	0.75 ± 0.23	0.73 ± 0.23	0.135	0.72 ± 0.25	0.68 ± 0.20	0.159
Mean aortic valve gradient, mm Hg	40.2 ± 17.1	39.9 ± 16.7	42.0 ± 19.2	0.067	39.5 ± 16.8	41.0 ± 16.5	0.077	40.8 ± 18.1	44.7 ± 21.3	0.119
Left ventricular ejection fraction, %	55.1 ± 13.4	55.0 ± 13.4	55.7 ± 13.6	0.418	54.1 ± 13.7	57.3 ± 12.3	<0.001	54.7 ± 14.2	58.1 ± 11.7	0.064
Moderate or severe aortic regurgitation	163 (7.8%)	145 (8.1%)	18 (6.4%)	0.404	111 (8.7%)	34 (6.6%)	0.152	12 (6.1%)	6 (7.1%)	0.793
Moderate or severe mitral regurgitation	359 (19.1%)	314 (19.3%)	45 (17.6%)	0.607	222 (19.0%)	92 (20.0%)	0.676	31 (17.1%)	14 (18.9%)	0.721
Procedural characteristics										
General anesthesia	234 (11.2%)	199 (11.1%)	35 (12.4%)	0.543	150 (11.7%)	49 (9.4%)	0.184	25 (12.6%)	10 (11.8%)	1.00
Valve type	(N = 2,080)	(n = 1,799)	(n = 281)	<0.001	(n = 1,280)	(n = 519)	0.968	(n = 196)	(n = 85)	0.887
Balloon expandable	1,074 (51.6%)	955 (53.1%)	119 (42.3%)	0.001	681 (53.2%)	274 (52.8%)	0.876	84 (42.9%)	35 (41.2%)	0.896
Self-expanding	870 (41.8%)	747 (41.5%)	123 (43.8%)	0.475	531 (41.5%)	216 (41.6%)	0.958	84 (42.9%)	39 (45.9%)	0.695
Mechanically expandable	136 (6.5%)	97 (5.4%)	39 (13.9%)	<0.001	68 (5.3%)	29 (5.6%)	0.818	28 (14.3%)	11 (12.9%)	0.852
Device generation	(N = 2,080)	(n = 1,799)	(n = 281)	0.414	(n = 1,280)	(n = 519)	0.949	(n = 196)	(n = 85)	0.570
Earlier-generation	594 (28.6%)	508 (28.2%)	86 (30.6%)	0.435	362 (28.3%)	146 (28.1%)	1.00	62 (31.6%)	24 (28.2%)	0.673
Newer-generation	1,486 (71.4%)	1,291 (71.8%)	195 (69.4%)	0.435	918 (71.7%)	373 (71.9%)	1.00	134 (68.4%)	61 (71.8%)	0.673
Valve size, mm	26.5 ± 2.3	26.5 ± 2.3	26.7 ± 2.3	0.290	26.5 ± 2.3	26.5 ± 2.2	0.832	26.7 ± 2.3	26.5 ± 2.2	0.312
Procedural outcomes										
Technical success	1,782 (85.5%)	1,547 (85.9%)	235 (83.0%)	0.203	1,098 (85.7%)	449 (86.5%)	0.708	170 (85.9%)	65 (76.5%)	0.059
Valve dislocation/embolization	36 (1.7%)	28 (1.6%)	8 (2.8%)	0.138	26 (2.0%)	2 (0.4%)	0.010	4 (2.0%)	4 (4.7%)	0.247
Conversion to surgical aortic valve replacement	13 (0.6%)	5 (0.3%)	8 (2.8%)	<0.001	4 (0.3%)	1 (0.2%)	1.00	5 (2.5%)	3 (3.5%)	0.70
Unplanned intervention related to cardiac structural complication^a^	29 (1.4%)	18 (1.0%)	11 (3.9%)	0.001	14 (1.1%)	4 (0.8%)	0.794	6 (3.0%)	5 (5.9%)	0.315
Stent placement for vascular/access-related complication	229 (11.0%)	201 (11.2%)	28 (9.9%)	0.609	139 (10.9%)	62 (11.9%)	0.509	17 (8.6%)	11 (12.9%)	0.281
Vascular surgery for vascular/access-related complication	13 (0.6%)	12 (0.7%)	1 (0.4%)	1.00	7 (0.5%)	5 (1.0%)	0.344	0	1 (1.2%)	0.30
Moderate or severe paravalvular regurgitation, n (%)	80 (3.8%)	68 (3.8%)	12 (4.2%)	0.739	48 (3.8%)	20 (3.9%)	0.892	9 (4.5%)	3 (3.5%)	1.00

Values are mean ± SD (*P* values from ANOVAs) or n (%) (*P* values from Fisher's test [2 × 2] or chi-square tests).

ANOVA = analysis of variance; CKD = chronic kidney disease; eGFR = estimated glomerular filtration rate; RAS = renin-angiotensin system; STS-PROM = Society of Thoracic Surgeons Predicted Risk of Mortality; TAVR = transcatheter aortic valve replacement; VARC = Valve Academic Research Consortium.

a including conversion to surgery, pericardial drainage due to annular rupture, and percutaneous coronary intervention due to coronary obstruction.

Table 1 shows baseline and procedural characteristics according to VARC-3 periprocedural myocardial injury and RAS inhibitor prescription. There were no significant differences in age, STS-PROM, and advanced heart failure symptoms (NYHA), baseline systolic blood pressure ≤100 mm Hg at baseline between patients with versus without RAS inhibitors. RAS inhibitor prescription was associated with a higher prevalence of comorbidities and lower left ventricular ejection fraction.

### Clinical outcomes

**Figure 2:**
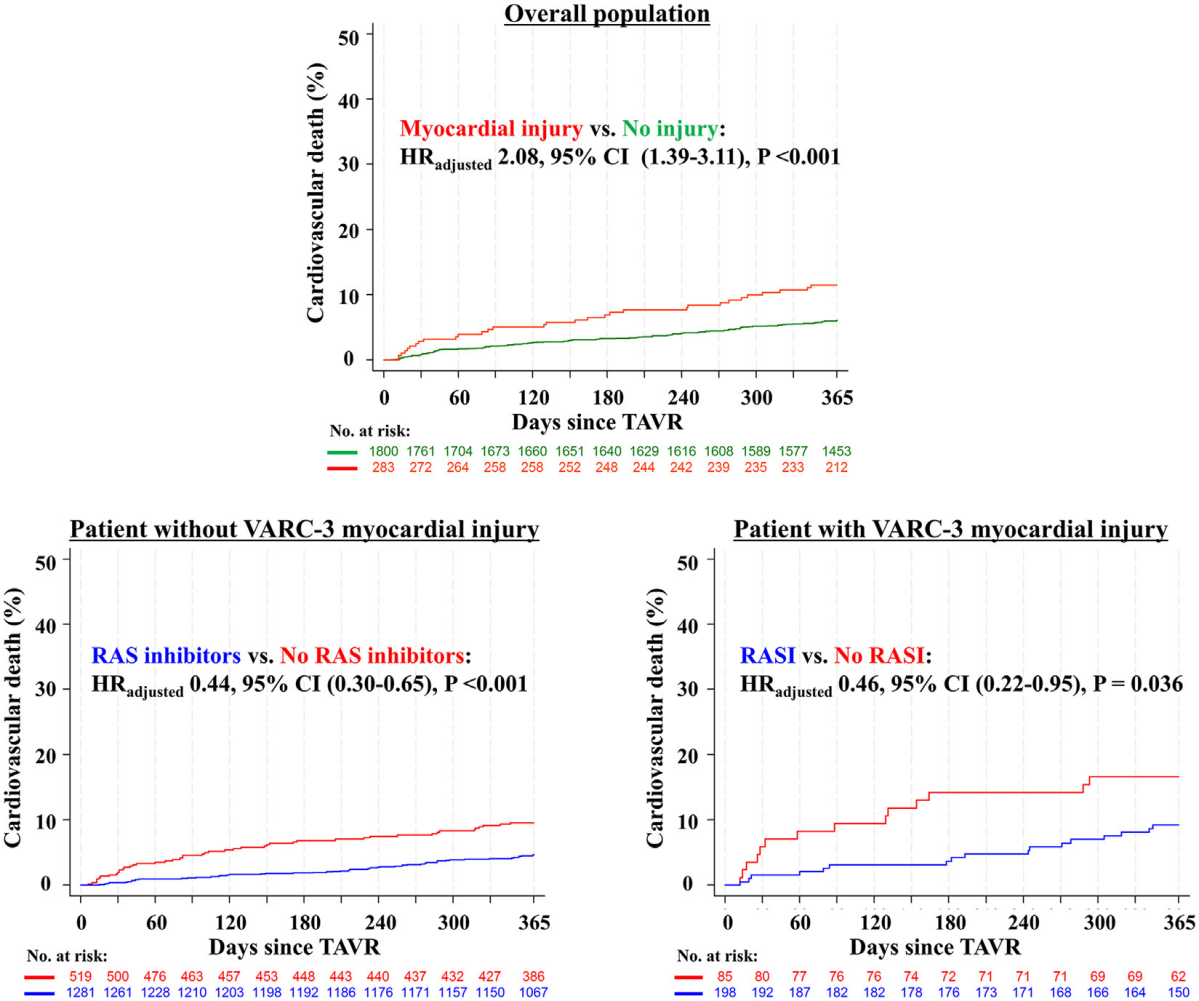
**Kaplan-Meier Curves for Cardiovascular Death According to VARC-3 Periprocedural Myocardial Injury and RAS Inhibitor Prescription at Discharge** RASI = renin-angiotensin system inhibitors; VARC = Valve Academic Research Consortium; other abbreviations as in Figure 1.

Clinical outcomes at 30 days and 1 year after TAVR are summarized in Table 2. Compared with patients without periprocedural myocardial injury, those with myocardial injury had an increased risk of cardiovascular death at 30 days and 1 year after TAVR (HR_adjusted:_ 3.37; 95% CI: 1.42-7.99; *P* = 0.006 and HR_adjusted:_ 2.08; 95% CI: 1.39-3.11; *P* < 0.001, respectively) (Figure 2).

**Table 2:** Clinical Outcomes According to VARC-3 Periprocedural Myocardial Injury and RAS Inhibitor Prescription at Discharge

	Overall Population						Patients Without Myocardial Injury						Patients With Myocardial Injury					
	Patients Without Myocardial Injury (N = 1,800)	Patients With Myocardial Injury (N = 283)	Myocardial Injury vs No Injury				RAS Inhibitors (n = 1,281)	No RAS Inhibitors (n = 519)	RAS Inhibitors vs No RAS Inhibitors				RAS Inhibitors (n = 198)	No RAS Inhibitors (n = 85)	RAS Inhibitors vs No RAS Inhibitors			
			HR (95% CI)	*P* Value	Adjusted HR (95% CI)	Adjusted *P* Value			HR (95% CI)	*P* Value	Adjusted HR (95% CI)	Adjusted *P* Value			HR (95% CI)	*P* Value	Adjusted HR (95% CI)	Adjusted *P* Value
30-day outcomes																		
Cardiovascular mortality	15 (0.8%)	8 (2.9%)	3.43 (1.45-8.09)	0.005	3.37 (1.42-7.99)	0.006	5 (0.4%)	10 (2.0%)	0.20 (0.07-0.58)	0.003	0.19 (0.07-0.57)	0.003	3 (1.5%)	5 (5.9%)	0.26 (0.06-1.08)	0.063	0.22 (0.05-0.96)	0.044
NYHA functional III or IV	139/1,675 (8.3%)	24/254 (9.4%)	1.14 (0.75-1.72)	0.538	1.14 (0.75-1.72)	0.534	87/1,207 (7.2%)	52/468 (11.1%)	0.65 (0.47-0.90)	0.009	0.64 (0.46-0.89)	0.009	18/179 (10.1%)	6/75 (8.0%)	1.26 (0.52-3.05)	0.613	1.39 (0.56-3.44)	0.476
1-year outcomes																		
Cardiovascular mortality^a^	100 (5.9%)	31 (11.6%)	2.03 (1.36-3.04)	0.001	2.08 (1.39-3.11)	<0.001	54 (4.5%)	46 (9.5%)	0.45 (0.30-0.66)	<0.001	0.44 (0.30-0.65)	<0.001	17 (9.4%)	14 (16.6%)	0.52 (0.25-1.05)	0.068	0.46 (0.22-0.95)	0.036
NYHA functional III or IV	161/1,528 (10.5%)	27/225 (12.0%)	1.14 (0.78-1.67)	0.506	1.12 (0.76-1.65)	0.567	114/1,113 (10.2%)	47/415 (11.3%)	0.90 (0.66-1.25)	0.539	0.88 (0.64-1.22)	0.451	18/157 (11.5%)	9/68 (13.2%)	0.87 (0.41-1.83)	0.707	0.83 (0.40-1.74)	0.624

Cox's time to first event regressions with HR (95% CI) and Wald *P* values reported. Only first event/patient considered and percentages from Kaplan-Meier estimates (%).

NYHA functional class III or IV analyzed with robustified Poisson regressions, rate ratios with 95% confidence intervals and chi-square tests.

Adjusted hazard ratio with adjusted *P* values after adjustment for age, sex, body mass index (BMI) (single imputation with the mean for 6 missing BMI values), STS-PROM score.

Abbreviations as in Table 1, Table 2.

a Patients which died during the procedure are excluded.

Table 2 summarizes clinical outcomes at 30 days and 1 year after TAVR stratified by RAS inhibitor prescription and VARC-3 periprocedural myocardial injury. At 30 days, patients with RAS inhibitor prescription at discharge had a reduced risk of cardiovascular death compared with those without prescription in both the no myocardial injury group and the injury group (HR_adjusted:_ 0.19; 95% CI: 0.07-0.57; *P* = 0.003 and HR_adjusted:_ 0.22; 95% CI: 0.05-0.96; *P* = 0.044, respectively). At 1 year after TAVR, cardiovascular death occurred in 4.5% of patients receiving RAS inhibitors without periprocedural myocardial injury, in 9.5% of patients receiving no RAS inhibitors without myocardial injury, in 9.4% of patients receiving RAS inhibitors with myocardial injury, and in 16.6% of patients receiving no RAS inhibitors with myocardial injury (Figure 2). After adjustment for differences in baseline characteristics, the use of RAS inhibitors after TAVR was associated with a reduced risk of cardiovascular death in patients with and without periprocedural myocardial injury (HR_adjusted:_ 0.46; 95% CI: 0.22-0.95; *P* = 0.036, and HR_adjusted:_ 0.44; 95% CI: 0.30-0.65; *P* < 0.001, respectively). There was no significant difference in residual heart failure symptoms (NYHA functional class III or IV) between patients with and without RAS inhibitors in both groups.

### Full factorial model

In the full factorial model, there were significant differences in the risk of 1-year cardiovascular death between RAS inhibitor prescription and no prescription (HR: 0.81; 95% CI: 0.68-0.97; *P* = 0.021) and the development of periprocedural myocardial injury and no injury (HR: 1.58; 95% CI: 1.09-2.28; *P* = 0.015). There was no significant interaction between the development of periprocedural myocardial injury and RAS inhibitor prescription after TAVR for cardiovascular mortality (interaction HR: 0.75; 95% CI: 0.48-1.18; interaction *P* = 0.208) (Central Illustration).

### Sensitivity analysis

In the sensitivity analysis of 1,782 patients with technical success, periprocedural myocardial injury was observed in 235 patients (13.2%), and RAS inhibitors were prescribed in 170 patients (72.3%) with periprocedural myocardial injury and in 1,098 patients (71.0%) without injury. At 1 year after TAVR, patients with periprocedural myocardial injury had an increased risk of cardiovascular death (HR_adjusted:_ 1.74; 95% CI: 1.10-2.76; *P* = 0.018) compared with those without injury, and RAS inhibitor prescription after TAVR was associated with a reduced risk of cardiovascular death in patients with and without periprocedural myocardial injury (HR_adjusted:_ 0.44; 95% CI: 0.19-1.04; *P* = 0.060, and HR_adjusted:_ 0.50; 95% CI: 0.32-0.76; *P* < 0.001, respectively), consistent with the main analysis (Table 3).

**Table 3:** Clinical Outcomes in Patients With VARC-3 Technical Success

	Overall Population						Patients Without Myocardial Injury						Patients With Myocardial Injury					
	Patients Without Myocardial Injury (N = 1,547)	Patients With Myocardial Injury (N = 235)	Myocardial Injury vs No Injury				RAS Inhibitors (n = 1,098)	No RAS Inhibitors (n = 449)	RAS Inhibitors vs No RAS Inhibitors				RAS Inhibitors (n = 170)	No RAS Inhibitors (n = 65)	RAS Inhibitors vs No RAS Inhibitors			
			HR (95% CI)	*P* Value	Adjusted HR (95% CI)	Adjusted *P* Value			HR (95% CI)	*P* Value	Adjusted HR (95% CI)	Adjusted *P* Value			HR (95% CI)	*P* Value	Adjusted HR (95% CI)	Adjusted *P* Value
30-day outcomes																		
Cardiovascular mortality	13 (0.8%)	4 (1.7%)	2.03 (0.66-6.24)	0.214	1.85 (0.60-5.76)	0.287	4 (0.4%)	9 (2.0%)	0.18 (0.06-0.58)	0.004	0.17 (0.05-0.57)	0.004	2 (1.2%)	2 (3.1%)	0.39 (0.05-2.74)	0.342	0.29 (0.04-2.27)	0.237
NYHA functional III or IV	121/1,440 (8.4%)	20/215 (9.3%)	1.11 (0.71-1.74)	0.659	1.10 (0.70-1.73)	0.680	77/1,037 (7.4%)	44/403 (10.9%)	0.68 (0.48-0.97)	0.032	0.68 (0.47-0.96)	0.030	17/157 (10.8%)	3/58 (5.2%)	2.09 (0.64-6.90)	0.225	2.21 (0.65-7.54)	0.206
1-year outcomes																		
Cardiovascular mortality^a^	87 (6.0%)	23 (10.4%)	1.77 (1.12-2.80)	0.015	1.74 (1.10-2.76)	0.018	50 (4.9%)	37 (8.8%)	0.52 (0.34-0.80)	0.003	0.50 (0.32-0.76)	<0.001	13 (8.4%)	10 (15.6%)	0.50 (0.22-1.13)	0.097	0.44 (0.19-1.04)	0.060
NYHA functional III or IV	140/1,309 (10.7%)	23/188 (12.2%)	1.14 (0.76-1.73)	0.524	1.13 (0.75-1.72)	0.555	100/948 (10.5%)	40/361 (11.1%)	0.95 (0.67-1.35)	0.781	0.92 (0.65-1.30)	0.625	17/136 (12.5%)	6/52 (11.5%)	1.08 (0.45-2.60)	0.858	1.04 (0.44-2.42)	0.936

Cox's time to first event regressions with HR (95% CI) and Wald *P* values reported. Only first event/patient considered and percentages from Kaplan-Meier estimates (%).

NYHA functional class III or IV analyzed with robustified Poisson regressions, rate ratios with 95% confidence intervals and chi-square tests.

Adjusted hazard ratio with adjusted *P* values after adjustment for age, sex, body mass index (BMI) (single imputation with the mean for 6 missing BMI values), STS-PROM score.

Abbreviations as in Table 1.

^a^ Patients which died during the procedure are excluded.

### Exploratory analysis

**Figure 3:**
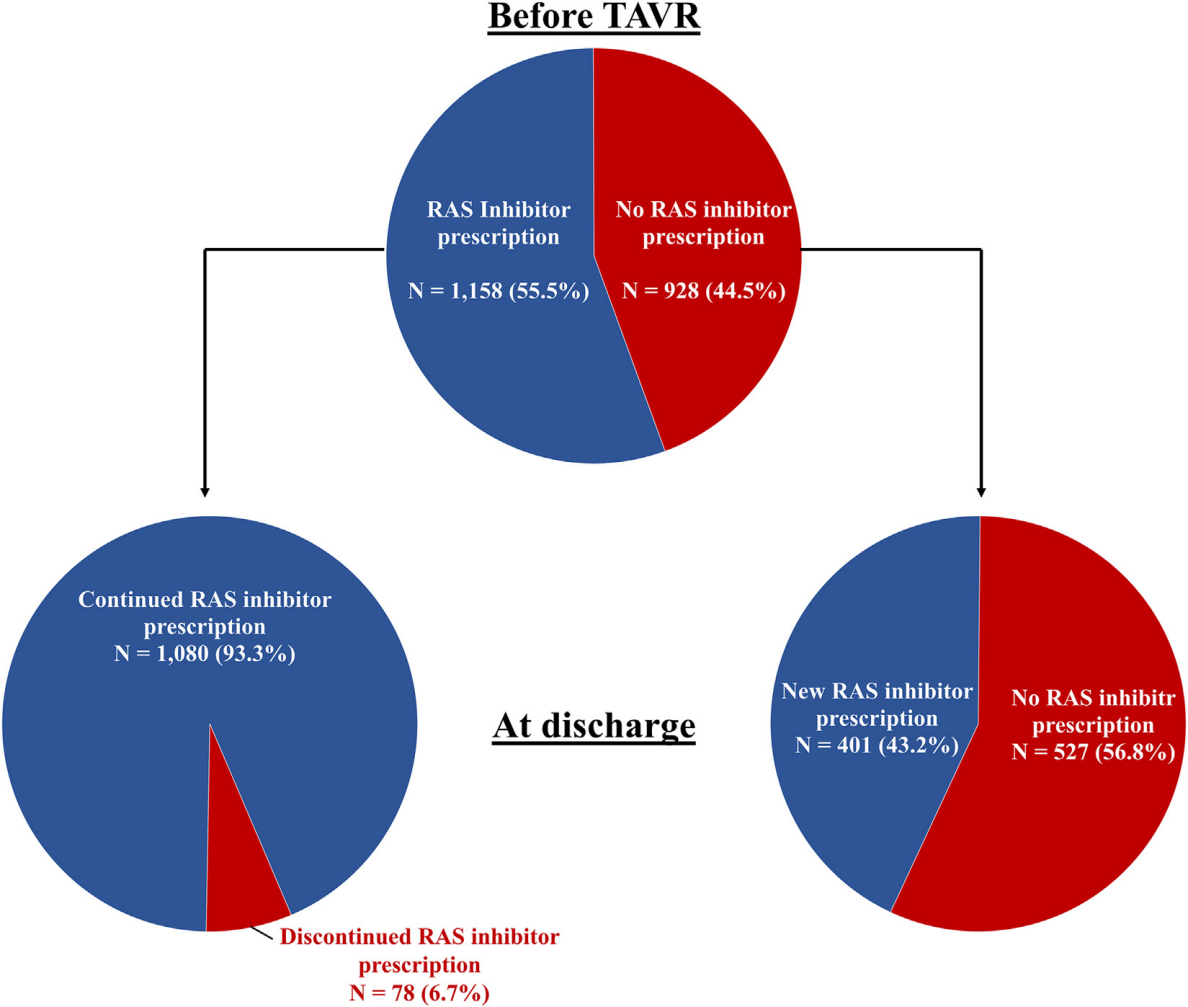
**Change in RAS Inhibitor Prescription After TAVR** Patients with information on RAS inhibitor prescription both at baseline and discharge and the assessment of VARC-3 periprocedural myocardial injury were included (N = 2,086). Abbreviations as in Figure 1.

To investigate the association between baseline RAS inhibitor prescription and the development and the prognostic association of VARC-3 periprocedural myocardial injury, we performed exploratory analyses according to baseline RAS inhibitor prescription. A total of 3,163 patients with the information on baseline RAS inhibitor prescription and post-TAVR cardiac biomarkers were included in the analysis. At baseline, 1,789 patients (56.6%) were prescribed RAS inhibitors. Baseline and procedural characteristics according to RAS inhibitor prescription at baseline and the development of VARC-3 periprocedural myocardial injury are shown in Supplemental Table 1. After TAVR, 8.0% and 7.3% of patients with and without RAS inhibitors, respectively, developed VARC-3 myocardial injury. In the logistic regression analysis, RAS inhibitor prescription was not associated with a reduced risk of developing VARC-3 periprocedural myocardial injury (adjusted OR: 1.12; 95% CI: 0.86-1.46; *P* = 0.406) (Table 4). In the outcome analysis, there was no difference in the incidence of cardiovascular mortality and the rate of NYHA III or IV at 1 year between patients with and without prescription of RAS inhibitors at baseline, regardless of the development of periprocedural myocardial injury (Supplemental Table 2, Supplemental Figure 1). At discharge, RAS inhibitors were discontinued in 78 patients (6.7%) in the group of the RAS inhibitor prescription at baseline and started in 401 patients (43.2%) in the group of no RAS inhibitor prescription at baseline (Figure 3).

**Table 4:** Relationship Between Baseline RAS Inhibitor Prescription and Development of VARC-3 Myocardial Injury

	RAS Inhibitors at Baseline	No RAS Inhibitors at Baseline	OR (95% CI)	*P* Value	Adjusted OR (95% CI)	Adjusted *P* Value
	(n = 1,789)	(n = 1,374)				
VARC-3 periprocedural myocardial injury	144 (8.0%)	100 (7.3%)	1.12 (0.86-1.45)	0.421	1.12 (0.86-1.46)	0.406

No. of events/n assessed (%). Logistic regressions with OR (95% CI) and Wald *P* values reported.

Abbreviations as in Table 1.

## Discussion

The salient findings of this study are as follows: 1) in a prospective TAVR registry, 1 out of 7 patients with severe AS developed VARC-3 periprocedural myocardial injury after TAVR; 2) VARC-3 periprocedural myocardial injury was associated with a 2-fold increased risk of cardiovascular death at 1 year; 3) 30% of patients were not prescribed RAS inhibitors after TAVR, irrespective of the development of myocardial injury; 4) prescription of RAS inhibitors was associated with a reduced risk of 1-year cardiovascular mortality after TAVR in both patients with and without development of periprocedural myocardial injury with no significant interaction between groups.

Periprocedural elevations in cardiac biomarkers after TAVR are common and result from a combination of several factors: 1) direct myocardial damage induced by mechanical compression of the left ventricular outflow tract during balloon dilatation and valve implantation; 2) distal embolization of calcium microparticles into the coronary arteries; and 3) myocardial supply-demand mismatch due to transient hypotension during rapid pacing, excessive bradycardia because of conduction abnormalities, and ischemia induced by inotropic hemodynamic support during the procedure.^4^^,^^24^ Previous studies have identified several patient characteristics as predictors of periprocedural myocardial injury, including female sex, hypertension, and peripheral artery disease.^5^^,^^7^^,^^9^ Similarly, procedural factors may influence the elevation of cardiac biomarkers. In the randomized DIRECTAVI (Direct Transcatheter Aortic Valve Implantation) trial comparing TAVR with contemporary balloon-expandable valves with versus without balloon predilatation, predilatation prior to valve deployment was an independent predictor of periprocedural myocardial injury.^7^ Procedural adverse events may also increase the risk of myocardial injury. In the present analysis, conversion to surgical aortic valve replacement was more frequent in patients with myocardial injury. This observation corroborates the findings of a previous study in which reported procedural time was the only predictor of myocardial injury.^5^ Of note, our data show that 13% of patients develop periprocedural myocardial injury even in the absence of procedural complications. In addition, preprocedural RAS inhibitors did not reduce the incidence of periprocedural myocardial injury after TAVR. These findings are consistent with the observations of previous studies. Preprocedural prescription of RAS inhibitors did not reduce the incidence of periprocedural myocardial injury in patients undergoing cardiac surgery.^25^^,^^26^ Different mechanisms of periprocedural elevations in cardiac biomarkers may outweigh the protective effect of RAS inhibitors in myocardial injury.^27^ Further studies are warranted to determine the optimal strategy to reduce the risk of periprocedural myocardial injury.

The definition of periprocedural myocardial injury in the VARC criteria has changed significantly over the past decade.^3^^,^^8^^,^^18^ Lower thresholds for cardiac biomarker elevations to define periprocedural myocardial increase the sensitivity of diagnosis but may overestimate the incidence of clinically relevant myocardial injury. Indeed, in previous studies, the incidence of periprocedural myocardial injury according to the VARC-2 definition ranged from 20 to 60%, and the prognostic implications were inconsistent.^4^, ^5^, ^6^, ^7^ A recent multicenter study including 1,394 patients undergoing transarterial TAVR reported that the use of the more specific VARC-3 definition resulted in a lower incidence of periprocedural myocardial injury as compared to the VARC-2 definition (14% vs 59%), and the occurrence of periprocedural myocardial injury according to the VARC-3 definition was associated with an increased risk of 1-year mortality and lack of improvement of left ventricular ejection fraction after TAVR.^9^ Consistent with this study by Real and colleagues, the incidence of VARC-3 periprocedural myocardial injury in the present study was 13.8%, and the occurrence of periprocedural myocardial injury was associated with a 2-fold increased risk of 1-year cardiovascular mortality after TAVR. These findings underscore the importance of dedicated strategies to optimize clinical outcome of patients with periprocedural myocardial injury.

RAS blocker therapy has proven effective across a large spectrum of cardiovascular disease.^1^^,^^2^^,^^28^, ^29^, ^30^, ^31^, ^32^ In AS patients undergoing TAVR, RAS inhibitors promote the reduction of cellular hypertrophy and myocardial fibrosis (in addition to the relief from pressure overload by AVR) and favorably impact prognosis.^12^^,^^13^^,^^33^^,^^34^ Similarly, in patients with acute myocardial infarction, RAS inhibition attenuates left ventricular remodeling and mitigates the risk of heart failure.^10^ Against these pathophysiological considerations, it can be hypothesized that the prescription of RAS inhibitors to patients with myocardial injury after TAVR may favorably affect prognosis. Interestingly, in our registry, only half of the patients received RAS inhibitors prior to TAVR, and there was no difference in clinical outcomes at 1 year between patients with and without prescription of RAS inhibitors at baseline, regardless of the development of periprocedural myocardial injury. However, these results may be confounded by the change in RAS inhibitor prescription after TAVR. Indeed, more than 40% of patients without RAS inhibitors at baseline were newly prescribed RAS inhibitors at discharge, and the incidence of cardiovascular death and the rate of NYHA functional class III or IV in patients without RAS inhibitors was relatively lower in the analysis based on the baseline RAS inhibitor prescription than in the analysis based on discharge RAS inhibitor prescription. In contrast, patients prescribed RAS inhibitors at discharge had improved survival at 1 year with a consistent effect in patients with and without periprocedural myocardial injury. Given that vasodilators may be considered unsafe and contraindicated in the setting of severe AS, initiation of RAS inhibitors after TAVR represents a reasonable option.^13^ Nevertheless, only 70% of patients were discharged on RAS inhibitors after TAVR irrespective of the development of periprocedural myocardial injury. Despite the proven benefits of RAS blocker therapy across a large spectrum of cardiovascular disease, considerable underutilization of RAS inhibitors remains a challenge in the elderly population.^11^^,^^12^ In the Euro Heart Failure Survey II, patients aged ≥80 years were less commonly prescribed heart failure medications than those <80 years of age. High prevalence of comorbidities, frailty, concerns about polypharmacy, and social circumstances may complicate adherence and explain the underutilization of heart failure medications in the elderly population.^35^^,^^36^ An individualized approach tailored to the needs and preferences of the patient, tolerance, side effects, and drug interactions remains key in this population.

It should be noted that the association of RAS prescription with lower cardiovascular mortality was consistent in TAVR patients with and without periprocedural myocardial injury, whereas the use of RAS inhibitors in patients with myocardial injury did not improve prognosis to the same extent as in patients without injury. Therefore, the present analysis of observational data does not indicate an accentuated benefit of RAS inhibition in this clinical setting and does not provide specific guidance for the management of patients with periprocedural myocardial injury. Further studies are needed to determine the optimal post-TAVR management strategies to improve the prognosis of patients with periprocedural myocardial injury.

### Study limitations

The findings of our study should be interpreted in light of several limitations. First, more than 1,000 patients were excluded because of incomplete information required for the assessment of VARC-3 periprocdural myocardial injury, which may have introduced a degree of selection bias. In turn, we provide comprehensive data on 2,000 patients with granular assessment of periprocdural myocardial injury from a large prospective registry with high data quality standards and independent event adjudication. Second, information on dose and adherence to RAS inhibitors during follow-up was unknown. In addition, we did not have information on the reasons why RAS inhibitors were or were not prescribed. Patients who were not expected to benefit from RAS inhibitors because of their short life expectancy, comorbidity, frailty, or intolerance may have been included in the present analysis. Given that healthier patients may be more likely to receive RAS inhibitors, caution should be warranted in interpreting the present results, as the beneficial effect of RAS inhibitors may be overestimated. Moreover, our registry only records medication status at baseline and discharge, and we were unable to adjust for change in the prescription status into account, which may introduce selection bias. Third, our registry does not collect heart failure hospitalization. However, we report NYHA functional classification, which allows the assessment of the health-related quality of life. Fourth, the present cohort included predominantly octogenarians, and the results may not be generalizable to younger patients with less comorbidities and longer life expectancy. Fifth, the results of the present study reflect the experience of a single high-volume center and may not be generalizable to other heart centers with a different patient population. Finally, as this was a retrospective analysis based on a prospective registry, the possibility of residual confounding cannot be excluded despite rigorous statistical techniques.

## Conclusions

In patients undergoing TAVR, 1 out of 7 patients developed periprocedural myocardial injury according to the recently defined VARC-3 definition. VARC-3 periprocedural myocardial injury was associated with a 2-fold increased risk of cardiovascular death at 1 year after TAVR. The favorable association of RAS inhibitor prescription was consistent in patients with and without periprocedural myocardial injury.

## Funding support and author disclosures

Dr Pilgrim has received research grants from the Swiss National Science Foundation, the Swiss Heart Foundation, the Swiss Polar Institute, and the Bangerter-Rhyner Foundation. Research; travel or educational grants to the institution without personal remuneration from Biotronik, Boston Scientific, Edwards Lifesciences, and ATSens; and speaker fees and consultancy fees to the institution from Biotronik, Boston Scientific, Edwards Lifesciences, Abbott, Medtronic, Biosensors, and Highlife. Dr Windecker has received research, travel, or educational grants to the institution without personal remuneration from Abbott, Abiomed, Amgen, AstraZeneca, Bayer, Braun, Biotronik, Boehringer Ingelheim, Boston Scientific, Bristol Myers Squibb, Cardinal Health, CardioValve, Cordis Medical, Corflow Therapeutics, CSL Behring, Daiichi Sankyo, Edwards Lifesciences, Farapulse Inc Fumedica, Guerbet, Idorsia, Inari Medical, InfraRedx, Janssen-Cilag, Johnson & Johnson, Medalliance, Medicure, Medtronic, Merck Sharp & Dohm, Miracor Medical, MonarQ, Novartis, Novo Nordisk, Organon, OrPha Suisse, and Pharming Tech. Pfizer, Polares, Regeneron, Sanofi-Aventis, Servier, Sinomed, Terumo, Vifor, V-Wave. He served as advisory board member and/or member of the steering/executive group of trials funded by 10.13039/100000046Abbott, 10.13039/100020297Abiomed, 10.13039/100002429Amgen, 10.13039/100004325AstraZeneca, 10.13039/100004326Bayer, 10.13039/100008497Boston Scientific, 10.13039/501100005035Biotronik, Bristol Myers Squibb, 10.13039/100006520Edwards Lifesciences, 10.13039/501100023518MedAlliance, 10.13039/100004374Medtronic, 10.13039/100004336Novartis, Polares, Recardio, Sinomed, 10.13039/501100008645Terumo, and V-Wave with payments to the institution but no personal payments and is also member of the steering/executive committee group of several investigator-initiated trials that receive funding by industry without impact on his personal remuneration. Dr Reineke reports travel expenses from Abbott, Edwards Lifesciences, and Medtronic. Dr Stortecky reports research grants to the institution from 10.13039/100006520Edwards Lifesciences, 10.13039/100004374Medtronic, 10.13039/100008497Boston Scientific, and 10.13039/100000046Abbott and personal fees from Boston Scientific, Teleflex, and BTG. Dr Lanz reports speaker fees to the institution from Edwards Lifesciences and Abbott and served as advisory board member for Abbott. Dr Samim received funding for an online course from 10.13039/100006520Edwards Lifesciences. Dr Heg reports and with Department of Clinical Research, University of Bern, which has a staff policy of not accepting honoraria or consultancy fees. However, DCR is involved in design, conduct, or analysis of clinical studies funded by not-for-profit and for-profit organizations. In particular, pharmaceutical and medical device companies provide direct funding to some of these studies. For an up-to-date list of our conflicts of interest see https://www.ctu.unibe.ch/research_projects/declaration_of_interest/index_eng.html. All other authors have reported that they have no relationships relevant to the contents of this paper to disclose.

Perspectives **COMPETENCY IN MEDICAL KNOWLEDGE:** A recent study suggested that periprocedural myocardial injury according to the VARC-3 criteria more accurately delineates the incidence and prognostic association of periprocedural myocardial injury. However, the optimal medical treatment strategy after periprocedural myocardial injury has not been established. In a prospective TAVR registry, VARC-3 periprocedural myocardial injury was documented in 1 out of 7 patients undergoing TAVR and was associated with a 2-fold increased risk of cardiovascular mortality at 1 year. Prescription of RAS inhibitors was associated with a reduced risk of 1-year mortality after TAVR. A non-negligible proportion of patients were not prescribed RAS inhibitors after TAVR even after development of periprocedural myocardial injury.

**TRANSLATIONAL OUTLOOK:** Further studies are warranted to determine the optimal treatment strategy of patients with severe aortic stenosis undergoing TAVR complicated by myocardial injury.
