# Supplementary material for: Renin-Angiotensin System Inhibition in Patients With Myocardial Injury Complicating Transcatheter Aortic Valve Replacement
Source: JACC Adv. 2024 Aug 16;3(9):101212. doi: 10.1016/j.jacadv.2024.101212 (PMC11381816; doi:10.1016/j.jacadv.2024.101212)

Supplementary Table 1. Baseline and procedural characteristics according to VARC-3 periprocedural myocardial injury and RAS inhibitor prescription at baseline

	Patients without myocardial injury				Patients with myocardial injury			
	All patients N = 2,919	RAS inhibitors N = 1,645	No RAS inhibitors N = 1,274	P value	All patients N = 244	RAS inhibitors N = 144	No RAS inhibitors N = 100	P value
Age, years	81.9 ± 6.3	81.6 ± 6.2	82.2 ± 6.4	0.009	81.9 ± 6.0	82.4 ± 5.8	81.3 ± 6.2	0.176
Female, n (%)	1383 (47.4%)	772 (46.9%)	611 (48.0%)	0.601	128 (52.5%)	69 (47.9%)	59 (59.0%)	0.092
Body mass index, kg/cm²	26.8 ± 5.3	27.3 ± 5.3	26.1 ± 5.3	<0.001	26.6 ± 5.8	26.9 ± 5.3	26.5 ± 6.5	0.546
STS-PROM, %	4.6 ± 3.7	4.6 ± 3.6	4.7 ± 3.9	0.405	5.0 ± 3.2	5.2 ± 3.1	4.8 ± 3.4	0.288
NYHA III or IV, (%)	1725 (59.1%)	959 (58.3%)	766 (60.1%)	0.324	170 (69.7%)	98 (68.1%)	72 (72.0%)	0.572
Urgent TAVI, n (%)	62 (2.1%)	32 (1.9%)	30 (2.4%)	0.518	5 (2.0%)	2 (1.4%)	3 (3.0%)	0.403
Systolic blood pressure <100 mmHg, n (%)	470 (18.7%)	239 (17.0%)	231 (20.9%)	0.013	36 (18.7%)	21 (18.6%)	15 (18.8%)	1.00
**Comorbidities**								
Hypertension, n (%)	2554 (87.5%)	1576 (95.8%)	978 (76.8%)	<0.001	209 (85.7%)	134 (93.1%)	75 (75.0%)	<0.001
Diabetes mellitus, n (%)	827 (28.3%)	516 (31.4%)	311 (24.4%)	<0.001	66 (27.0%)	46 (31.9%)	20 (20.0%)	0.041
CKD (eGFR <60 mL/min/1.73 m²), n (%)	1896 (65.0%)	1049 (63.8%)	847 (66.5%)	0.127	168 (68.9%)	99 (68.8%)	69 (69.0%)	1.00
Coronary artery disease, n (%)	1602 (54.9%)	987 (60.0%)	615 (48.3%)	<0.001	148 (60.7%)	99 (68.8%)	49 (49.0%)	0.002

Previous myocardial infarction, n (%)	378 (12.9%)	249 (15.1%)	129 (10.1%)	<0.001	40 (16.4%)	33 (22.9%)	7 (7.0%)	0.001
Atrial fibrillation, n (%)	970 (33.2%)	529 (32.2%)	441 (34.6%)	0.166	84 (34.4%)	47 (32.6%)	37 (37.0%)	0.496
Peripheral artery disease, n (%)	255 (8.7%)	158 (9.6%)	97 (7.6%)	0.064	20 (8.2%)	18 (12.5%)	2 (2.0%)	0.003
**Echocardiography**								
Aortic valve area, cm²	0.77 ± 0.24	0.79 ± 0.23	0.74 ± 0.24	<0.001	0.73 ± 0.23	0.75 ± 0.24	0.69 ± 0.22	0.039
Mean aortic valve gradient, mmHg	39.3 ± 16.3	38.0 ± 15.8	40.9 ± 16.7	<0.001	41.4 ± 19.0	38.7 ± 17.4	45.3 ± 20.7	0.008
Left ventricular ejection fraction, %	55.5 ± 13.2	55.3 ± 13.3	55.7 ± 13.0	0.450	56.0 ± 13.0	54.6 ± 13.6	58.0 ± 12.1	0.049
Moderate or severe aortic regurgitation, n (%)	232 (8.0%)	131 (8.0%)	101 (7.9%)	1.00	16 (6.6%)	8 (5.6%)	8 (8.0%)	0.446
Moderate or severe mitral regurgitation, n (%)	472 (18.8%)	248 (17.6%)	224 (20.4%)	0.072	39 (17.5%)	20 (14.9%)	19 (21.3%)	0.280
**Procedural characteristics**								
General anesthesia, n (%)	291 (10.0%)	160 (9.7%)	131 (10.3%)	0.619	28 (11.5%)	15 (10.4%)	13 (13.0%)	0.546
Valve type, n (%)	N = 2916	N = 1644	N = 1272	0.411	N = 242	N = 142	N = 100	0.141
Balloon-expandable	1660 (56.9%)	942 (57.3%)	718 (56.4%)	0.651	99 (40.9%)	65 (45.8%)	34 (34.0%)	0.084
Self-expanding	1154 (39.6%)	651 (39.6%)	503 (39.5%)	1.00	106 (43.8%)	59 (41.5%)	47 (47.0%)	0.431
Mechanically-expandable	102 (3.5%)	51 (3.1%)	51 (4.0%)	0.188	37 (15.3%)	18 (12.7%)	19 (19.0%)	0.206
Device generation, n (%)	N = 2916	N = 1644	N = 1272	0.179	N = 242	N = 142	N = 100	0.621
Earlier-generation	427 (14.6%)	228 (13.9%)	199 (15.6%)	0.187	78 (32.2%)	44 (31.0%)	34 (34.0%)	0.676
Newer-generation	2489 (85.4%)	1416 (86.1%)	1073 (84.4%)	0.187	164 (67.8%)	98 (69.0%)	66 (66.0%)	0.676
Valve size, mm	26.4 ± 2.2	26.3 ± 2.2	26.6 ± 2.3	<0.001	26.78 ± 2.25	26.87 ± 2.33	26.66 ± 2.13	0.484

**Procedural outcomes**								
Technical success, n (%)	2599 (89.0%)	1454 (88.4%)	1145 (89.9%)	0.210	206 (84.4%)	122 (84.7%)	84 (84.0%)	1.00
Valve dislocation/embolization	31 (1.1%)	20 (1.2%)	11 (0.9%)	0.467	6 (2.5%)	3 (2.1%)	3 (3.0%)	0.691
Conversion to surgical aortic valve replacement	15 (0.5%)	6 (0.4%)	9 (0.7%)	0.296	3 (1.2%)	3 (2.1%)	0 (0.0%)	0.271
Unplanned intervention related to cardiac structural complication*	34 (1.2%)	18 (1.1%)	16 (1.3%)	0.730	4 (1.6%)	3 (2.1%)	1 (1.0%)	0.646
Stent placement for vascular/access-related complication	242 (8.3%)	143 (8.7%)	99 (7.8%)	0.380	25 (10.2%)	15 (10.4%)	10 (10.0%)	1.00
Vascular surgery for vascular/access-related complication	21 (0.7%)	13 (0.8%)	8 (0.6%)	0.664	1 (0.4%)	0 (0.0%)	1 (1.0%)	0.410
Moderate or severe paravalvular regurgitation, n (%)	80 (2.7%)	40 (2.4%)	40 (3.1%)	0.255	12 (4.9%)	8 (5.6%)	4 (4.0%)	0.766

Depicted are means with standard deviation (±), p-values from ANOVAs; counts with percentages (%), p-values from Fisher's test (2 x 2) or chi-square tests.

*including conversion to surgery, pericardial drainage due to annular rupture, and percutaneous coronary intervention due to coronary obstruction.

Abbreviations as in Table 1.

Supplementary Table 2. Clinical outcomes according to VARC-3 periprocedural myocardial injury and RAS inhibitor prescription at baseline

	Patients without myocardial injury						Patients with myocardial injury					
	RAS inhibitors N = 1,645	No RAS inhibitors N = 1,274	RAS inhibitors vs. No RAS inhibitors				RAS inhibition N = 144	No RAS inhibitors N = 100	RAS inhibitors vs. No RAS inhibitors			
			Hazard ratio (95% CI)	P value	Adjusted Hazard ratio (95% CI)	Adjusted P value			Hazard ratio (95% CI)	P value	Adjusted Hazard ratio (95% CI)	Adjusted P value
**30-day outcomes**												
Cardiovascular mortality, n (%)	25 (1.5%)	30 (2.4%)	0.64 (0.38-1.10)	0.105	0.68 (0.40-1.17)	0.163	5 (3.5%)	2 (2.0%)	1.75 (0.34-9.01)	0.505	1.47 (0.27-7.93)	0.655
NYHA III or IV, n (%)	98/1539 (6.4%)	90/1167 (7.7%)	0.83 (0.63-1.09)	0.174	0.82 (0.62-1.09)	0.171	16/127 (12.6%)	5/91 (5.5%)	2.29 (0.87-6.05)	0.093	2.37 (0.89-6.32)	0.085
**1-year outcomes**												
Cardiovascular mortality, n (%)	90 (6.2%)	89 (7.9%)	0.78 (0.58-1.05)	0.097	0.83 (0.61-1.11)	0.205	14 (10.3%)	10 (10.5%)	1.00 (0.44-2.26)	0.997	1.15 (0.50-2.68)	0.741
NYHA III or IV, n (%)	121/1314 (9.2%)	91/1001 (9.1%)	1.01 (0.78-1.31)	0.923	0.98 (0.75-1.27)	0.868	16/113 (14.2%)	9/83 (10.8%)	1.31 (0.61-2.81)	0.496	1.12 (0.53-2.39)	0.768

Cox's time to first event regressions with hazard ratios (95% confidence interval) and Wald p-values reported. Only first event/patient considered and percentages from Kaplan-Meier estimates (%). Administrative censoring at 1 year, at 30 days for TAVI performed 1 july 2022 and later.

NYHA III or IV analysed with robustified Poisson regressions, rate ratios with 95% confidence intervals and chi-square tests.

Adjusted hazard ratio with adjusted p-values after adjustment for age, sex, body mass index (single imputation with the mean for n=6 missing body mass index values), and STS-PROM score.

Abbreviations as in **Tables 1-3**.

Supplementary Figure. Kaplan-Meier curves for cardiovascular death according to VARC-3 periprocedural myocardial injury and RAS inhibitor prescription at baseline

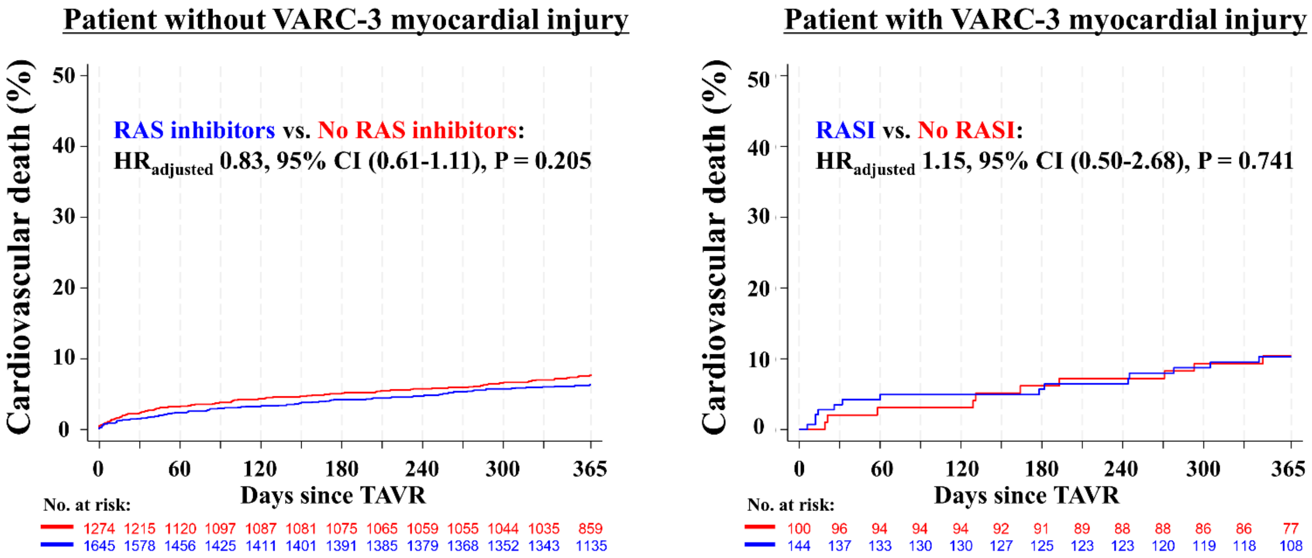

Supplement: Supplemental material [file mmc1.pdf]
